# Effects of dietary inorganic chromium supplementation on broiler growth performance: a meta-analysis

**DOI:** 10.7717/peerj.11097

**Published:** 2021-03-16

**Authors:** Chao Feng, Hua Lin, Jie Li, Bin Xie

**Affiliations:** 1 Department of Life Sciences, Hulunbuir University, Hulunbuir, Inner Mongolia Autonomous Region, China; 2 Department of Anesthesiology, Tianjin Medical University General Hospital Airport Site, Tianjin, China

**Keywords:** Inorganic chromium, Broiler, Growth performance, Meta analysis

## Abstract

**Background:**

A meta-analysis was conducted to assess dietary inorganic chromium supplementation on broiler growth performance and determine if these effects are regulated by strains, sex, or contextual factors such as study area and time.

**Methods:**

Eligible studies were identified by searching Web of Science, Springer, Elsevier, Science Direct, Taylor & Francis online databases. The weighted average difference with corresponding 95% confidence interval was computed with a random-effects model. We performed subgroup analyses stratified by study locations, published years, broiler sex, and strains. The publication bias was assessed with Egger’s test method. A total of nine studies were eligible for inclusion.

**Results:**

The meta-analysis results indicated that inorganic chromium supplementation significantly improved the broiler’s growth performance, with a lower feed conversion ratio (FCR) and a higher average daily feed intake (ADFI). Through subgroup analyses, we found that the result of average daily gain (ADG) in Iran or published in the 2010s, the results of ADFI in Egypt, and the results of FCR in China had significant responses to chromium supplementation. We also found that Cobb 500 broilers and male broilers might be more sensitive to the addition of inorganic chromium by subgroup analyses. A model was used to obtain the amount of chromium addition under the optimal growth performance. The results showed that the adjusted ADFI and FCR presented a quadratic relationship with chromium supplementation except for average daily gain (ADG). The growth performance improved when the inorganic chromium addition ranged from 1.6 to 2.3 mg/kg. The result of sensitivity analyses showed low sensitivity and high stability. Also, there was little indication of publication bias for studies.

**Conclusions:**

Our study showed that the males and Cobb 500 broilers might be more sensitive to chromium supplementation and provided more accurate inorganic chromium supplementation for broiler management practice. The fewer included studies may lead to higher heterogeneity, and no subgroup analyses of environmental stress conditions was conducted due to the lack of related information. Therefore, this study still has some limitations, and we look forward to the follow-up researches.

## Introduction

Chromium (Cr), as an essential trace element, plays a vital role in insulin action and promotes the efficiency of glucose, protein, and fat metabolism, which was also recognized as a dietary supplement by human beings (Haq et al., 2016). Chromium usually exists in organic and inorganic forms with different biological availability and absorption rates (Haq et al., 2016). Among inorganic sources, the most common forms of chromium are the metallic form (Cr^0^), trivalent form (Cr^3+^), and hexavalent form (Cr^6+^) (Haq et al., 2016). Trivalent chromium is the most stable oxidation state and is considered a highly safe form, whereas hexavalent chromium is a known toxin, mutagen, and carcinogen (Chowdhury et al., 2003).

Chromium is usually not considered an essential trace mineral in the broiler industry (Amata, 2013). However, studies showed that chromium could increase weight gain, feed conversion ratio, high density lipoprotein, and improve lean muscle development and nutrient digestion (Sahin, Sahin & Kucuk, 2003; Al-Bandr, Ibrahim & Al-Mashhadani, 2010). The beneficial effects of chromium can be observed more efficiently under environmental, dietary, and hormonal stress (Amata, 2013). Though currently there are no National Research Council (NRC, 1994) recommendations for chromium in broiler diets (National Research Council, 1994), lots of studies suggested that supplementation of chromium at different levels and combinations improved growth performance of broilers (Toghyani et al., 2012; Mohammed et al., 2014; Huang et al., 2016). Samanta, Haldar & Ghosh (2008) revealed that supplementation of CrCl_3_ at a dose of 0.5 mg/kg significantly improved the weight gain and food conversion ratio compared to the control group of birds, whereas Zheng et al. (2016) suggested that 0.4 mg/kg of CrCl_3_ had remarkable effects on carcass characteristics but not on growth performance. Some researchers also reported that average daily gain (ADG), average daily feed intake (ADFI) and, feed conversion ratio (FCR) were unaffected by CrCl_3_, Cr-yeast, or Cr picolinate (CrPic) treatments (Król et al., 2017; Lu et al., 2019).

Additionally, many studies indicated that chromium supplementation, regardless of its source in the broiler diet, positively affected growth performance and carcass traits under heat stress conditions  (Toghyani et al., 2012; Huang et al., 2016). According to Zha et al. (2009), inorganic chromium did not affect growth performance, but organic chromium did during heat stress conditions. Therefore, the effect of chromium addition, especially inorganic chromium addition, on broiler growth performance has not been concluded. However, chromium is still widely used as a common feed additive so far. In fact, there are two concerns regarding the effect of chromium supplementation. First, is adding chromium beneficial to growth performance? Second, what is the appropriate additive amount if it is beneficial?

Meta-analysis is a kind of statistical method that integrates research results of all types in the same field. The differences between studies are removed by meta-analysis, making the corrected data comparable, creating more objective and convincing conclusions (Sauvant et al., 2008). A meta-analysis entails consideration of the following aspects. (1) the results of included studies, (2) the comparisons of these results, (3) the effect of each comparison, (4) stability and reliability (Higgins & Cochrane Collaboration, 2020). Generally, meta-analysis is conducted to assess the strength of evidence present on treatment (Haidich, 2010), determine whether the effect is positive or negative, and further obtain the quantified estimate of the effect. The Cochrane Handbook provides an essential reference for meta-analysis (Higgins & Cochrane Collaboration, 2020). According to this handbook, the general steps are as follows: setting inclusion or exclusion criteria, retrieval process, data extraction, using models to estimate effect size, constructing forest plot, publication bias, sensitivity analyses, and subgroup analyses.

The main goal of this study is to explore the effect of inorganic chromium supplementation on broiler growth performance and the responses of different broiler sex and strains to chromium addition through meta-analysis. Moreover, a reasonable quantitative model needs to be built to explain the observed value and further provide a theoretical reference for the management practice of broiler.

## Materials & Methods

### Literature search strategy

All kinds of published studies were retrieved from the Web of Science, Springer, Elsevier, Science Direct, and Taylor & Francis online databases in October 2020. The following search terms were used (broiler OR chick*) AND (performance OR growth) AND (chromium chloride or inorganic chromium). Titles and abstracts of all potentially relevant publications were rigorously reviewed to assess their relevance to the study, and the full text was further scrutinized if any potentially relevant information was identified during the process.

### Inclusion/exclusion criteria

The selected studies met the following criteria: published in English; used a corn and soybean meal-based diet; had a 42-day experimental period; provided the specific inorganic chromium addition values; and pertained to non-indigenous breeds. Broiler chicks were divided into groups based on the inorganic chromium supply they received. Inorganic chromium addition in the control group was 0 mg/kg, while that in the experimental group ranged from 0.4 mg/kg to 4 mg/kg. During a 42-day experimental period, the selected research recorded live weight gain and daily food intake, and then calculated the ADG, ADFI, and FCR. The data in these studies included mean values, variances and encompassed at least three independent replicates of each treatment. These inclusion or exclusion criteria are basically the same as those used in our previous studies (Feng et al., 2020). Literature search and screening were performed independently by two reviewers (Chao Feng and Hua Lin), and any discrepancies will be decided by the third reviewer (Jie Li) or discussed to reach a consensus.

### Data extraction

Using a standardized data-collection protocol, we extracted the following data from each study: author name, country, published year, broiler strains, broiler sex, feed ingredients, inorganic chromium addition, sample size, experimental periods. We extracted the means, standard deviations (SD), and sample size (*n*) of ADG, ADFI, and FCR in both control and experiment treatment. When standard error (SE) was reported, we transformed it to SD by using the formula SD = SE * sqrt (*n*). If the data were presented graphically, we extracted data points through GetData software (http://www.getdata-graph-digitizer.com/). The above calculation and data extraction process are the same as those described in Feng et al. (2020). Data abstraction was performed independently by two reviewers (Chao Feng and Hua Lin), and any discrepancies will be decided by the third reviewer (Jie Li) or discussed to reach a consensus.

### Statistical analyses

The weighted average difference (WMD) is used to estimate the effect size of the combined study. This method used the pooled effect estimate to represent a weighted average of all included study group comparisons, where the weighting assigned to each study group comparison result is inversely proportional to the variance. This method assigns more weight in the meta-analysis to larger trials and less weight to the smaller ones (Cooper & Hedges, 1993). A random effects model taking into account both within and between study variation was assigned to compute the summary risk estimates (DerSimonian & Laird, 1986). We combined the data from studies and so WMDs with 95% confidence interval (CI) of a total change in ADG, ADFI, and FCR is the measure of the effect of interest in this meta-analysis. Stratified analyses by geographic areas, published year, broiler strains, and broiler sex were also carried out. To assess each study’s effects and verify the stability of the meta-analysis results, sensitivity analyses were also conducted by omitting each study in turn and estimating the overall effects of the remainder studies sequentially. Statistical heterogeneity was assessed with Q and *I*^2^ statistics (Higgins & Thompson, 2002). Potential publication bias was evaluated by using Egger’s test (Egger et al., 1997).

The PROC MIXED model (SAS Version 9.4; SAS Institute, Cary, NC) was used to analyze the relationship between inorganic chromium addition and broiler performance. The model is as follows:

\begin{eqnarray*}{Y}_{ij}={B}_{0}+{B}_{1}{X}_{ij}+{B}_{2}{X}_{ij}^{2}+{S}_{i}+{b}_{1i}{X}_{ij}+{b}_{2i}{X}_{ij}^{2}+{e}_{ij}. \end{eqnarray*}

The same mixed linear model was used in a previously published meta-analysis and was explained in details by Feng et al. (2020). The differences between the studies are assumed to be random effects. The intercept and slope of the variables (fixed effects) represent the average intercept and average slope of the ADG, ADFI, and FCR as they vary with dietary chromium additions in the mixed effects model (Feng et al., 2020). The intercept and slope of the variables (random effects) represent essential factors not included in the regression analyses in the different studies (Scheiner & Gurevitch, 2001). The random effects of the y value are adjusted to remove differences between the studies, and then a regression analysis is performed to calculate the correlation coefficient (i.e.,  *r*^2^) (St-Pierre, 2001). The mixed effects model code is shown below.

PROC MIXED data = data;

CLASS Group;

MODEL Y = X X*X/Solution OUTP = Predictionset OUTPM = PredY;

RANDOM intercept X/TYPE = VC SUBJECT = Group SOLUTION;

RUN;

A regression analysis and curve fitting were performed for the relationship between the variables (*y*-axis) and chromium addition (*x*-axis). The calculation steps were described in previous study by Feng et al. (2020). All the statistical analyses were performed using STATA (version 11.0; STATA Corp, College Station, TX) and SAS (Version 9.4; SAS Institute, Cary, NC) software.

## Results

### Selection process

**Figure 1:**
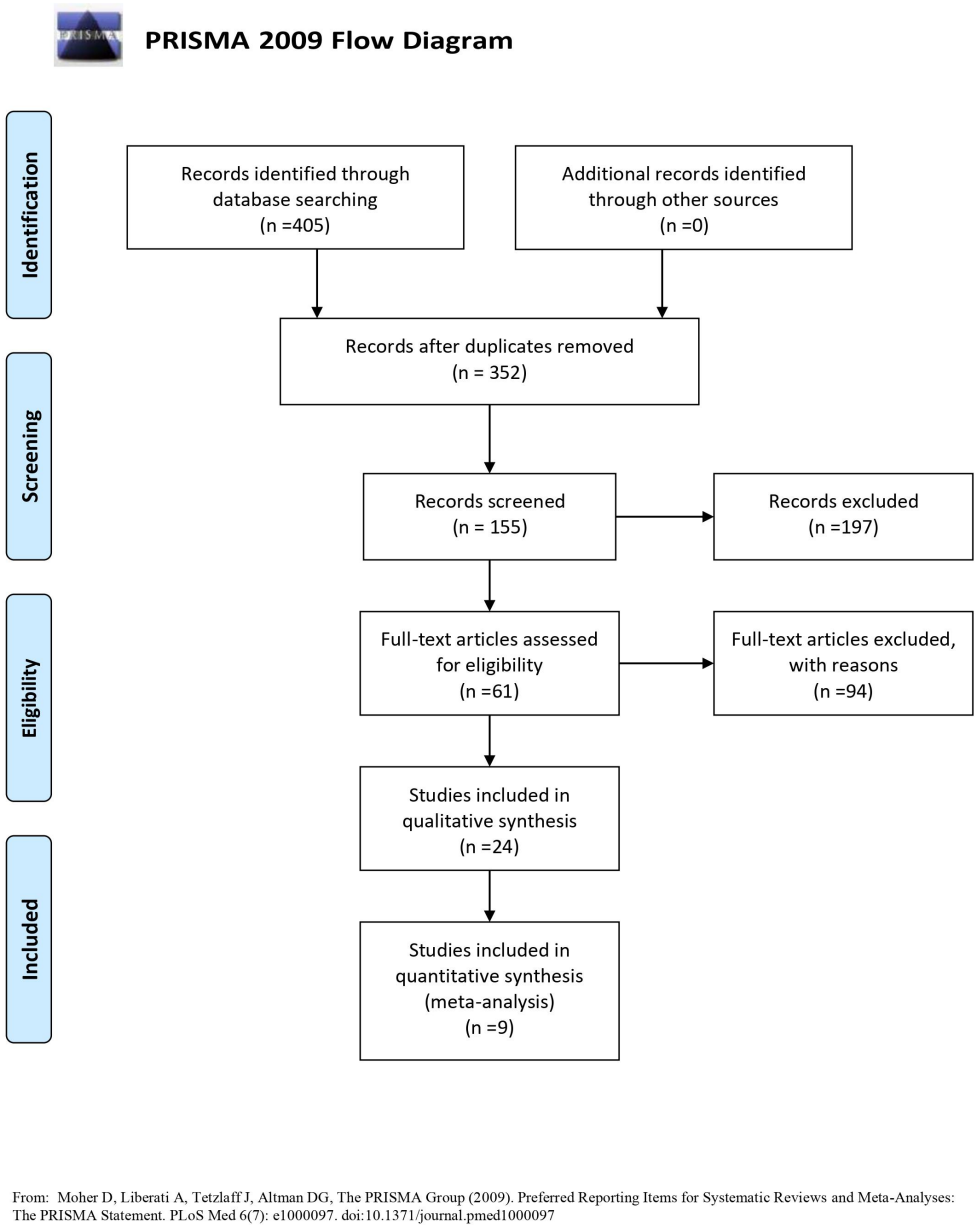
PRISMA flow diagram.

The search strategy yielded 405 citations (Fig. 1). No additional citations were found in references. After duplicates were removed, 352 full texts were retrieved. After carefully reading the title/abstract of these publications, irrelevant studies and studies that failed to report ADG, ADFI, FCR, or addition level were excluded. Finally, 9 studies were included in quantitative synthesis (meta-analysis).

### Study characteristics

We collected all the information related to this meta-analysis, the details of which were listed in Table S1. All the eligible studies were published between 2000 to 2020. Among them, 33.3% of the total studies were from 2000 to 2010, and 66.7% from 2000 to 2010. The study is mainly distributed in China, Iran, and Egypt, accounting for 44.4%, 33.3%, and 22.3%, respectively. The broiler strains included in the study were Cobb 500, Ross 308, Arbor Acres, and Hubbard, accounting for 33.3%, 33.3%, 22.2%, and 11.1%, respectively. Among the included studies, 4 studies used male broilers, 2 studies used female broilers, and the rest did not differentiate between sexes. Chromium addition ranged from 0.5 mg/kg to 3.2 mg/kg, and sample sizes ranged from 250 to 2700.

### Results of the meta-analysis

**Figure 2:**
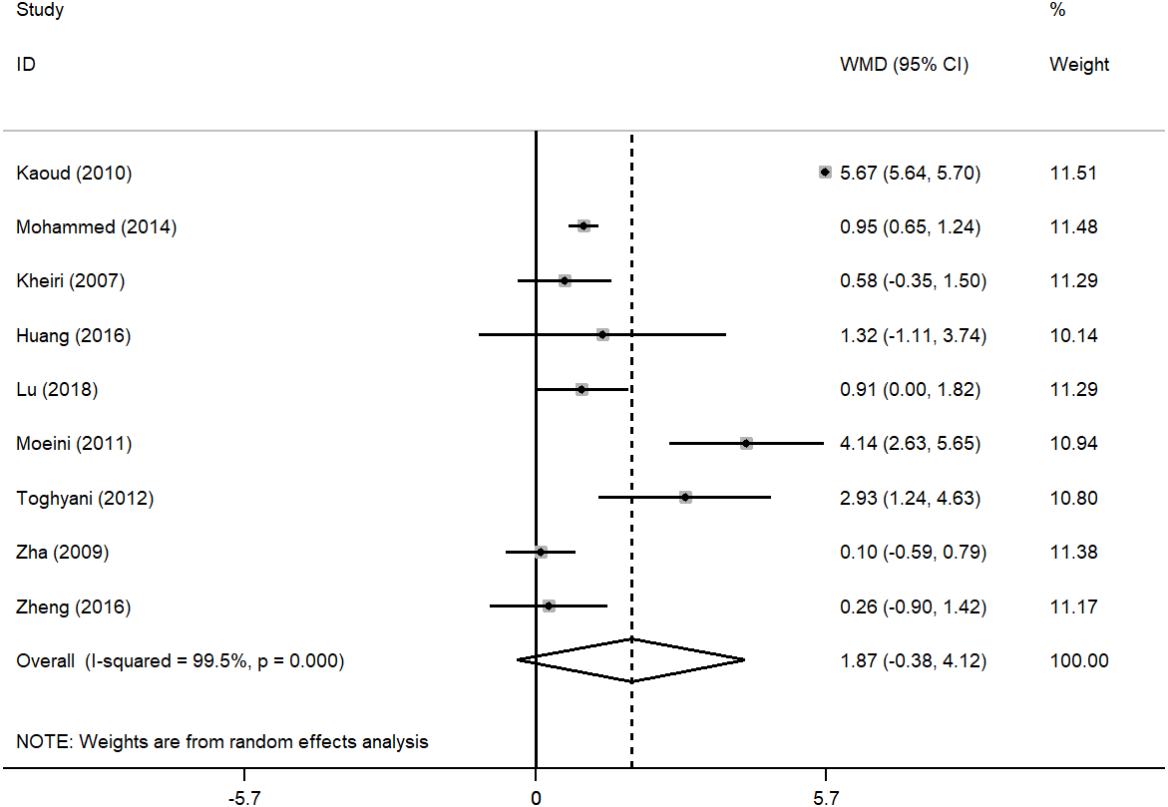
Forest plot of ADG.

**Figure 3:**
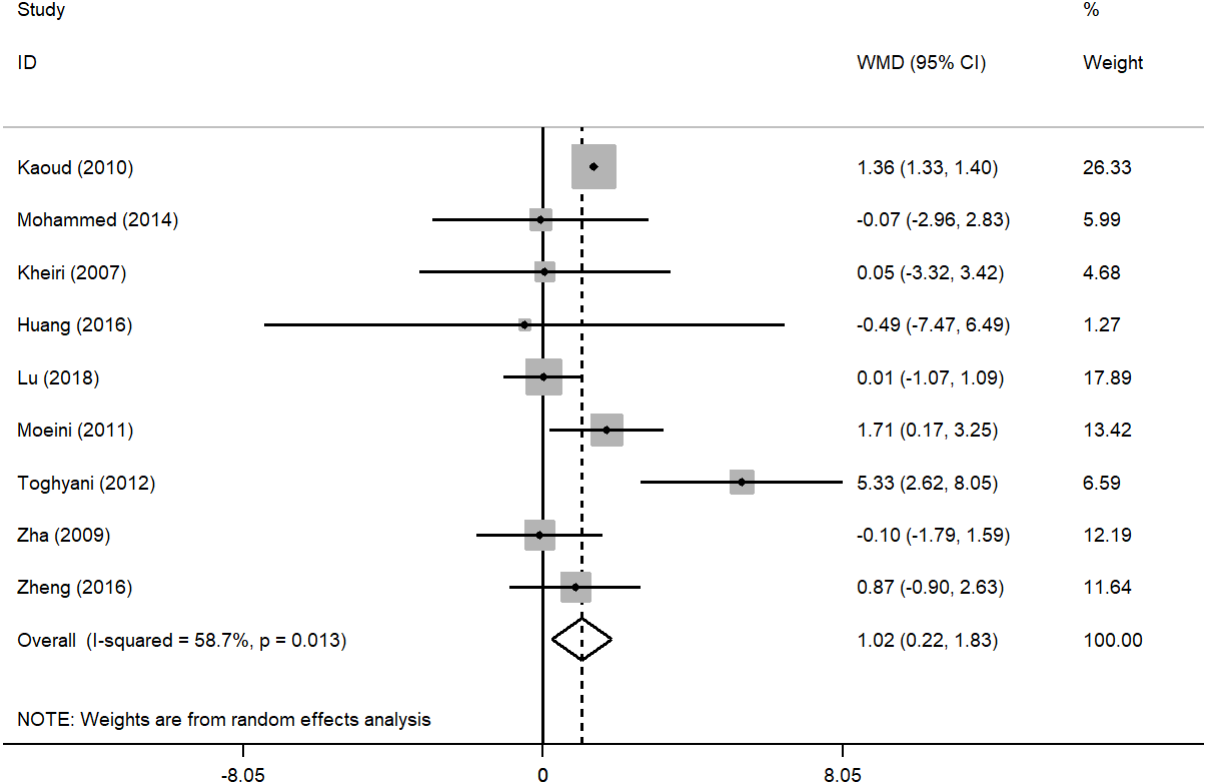
Forest plot of ADFI.

**Figure 4:**
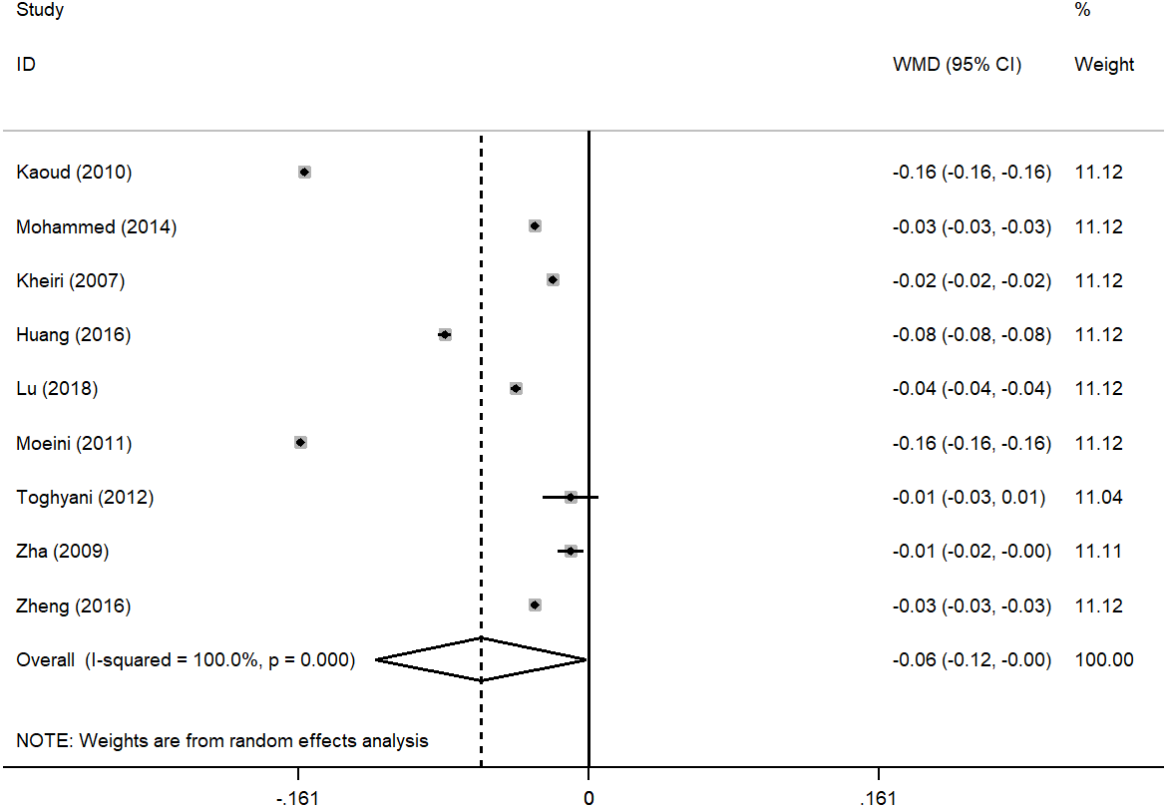
Forest plot of FCR.

Based on the results of the random effect method, the pooled WMDs of the ADG, ADFI, and FCR were 1.872 (95% CI [−0.376–4.121]), 1.023 (95% CI [0.217–1.828]), −0.060 (95% CI [−0.118 to −0.001]) (Figs. 2, 3 and 4), respectively. The addition of inorganic chromium in diet had no significant effect on ADG (*P* = 0.103), but had significant effect on ADFI and FCR (*P* = 0.013; *P* = 0.045) (Table 1). The subgroup analyses were stratified by study area, time, sex, strains. The pooled WMDs of ADG, ADFI, and FCR in different subgroups are listed in Table 1. We found that the results of ADG in Iran or published in the 2010s, the results of ADFI in Egypt, and the results of FCR in China had significant responses to chromium supplementation (*P* = 0.037, *P* = 0.001, *P* < 0.001, *P* = 0.001) (Table 1). The male broilers, Ross 308 and Cobb 500 showed obviously higher ADG (*P* = 0.047, *P* < 0.001, *P* = 0.037), and Cobb 500 also showed lower FCR than other broiler strains (*P* < 0.001). We detected significant heterogeneity in studies (*P* < 0.001, *P* = 0.013, *P* < 0.001) with *I*^2^ values ranging from 58.7% to 100% (Table 1). In general, heterogeneity was still present but was attenuated in ADFI compared with ADG and FCR (Table 1).

### Relationship between performance and inorganic chromium addition

**Figure 5:**
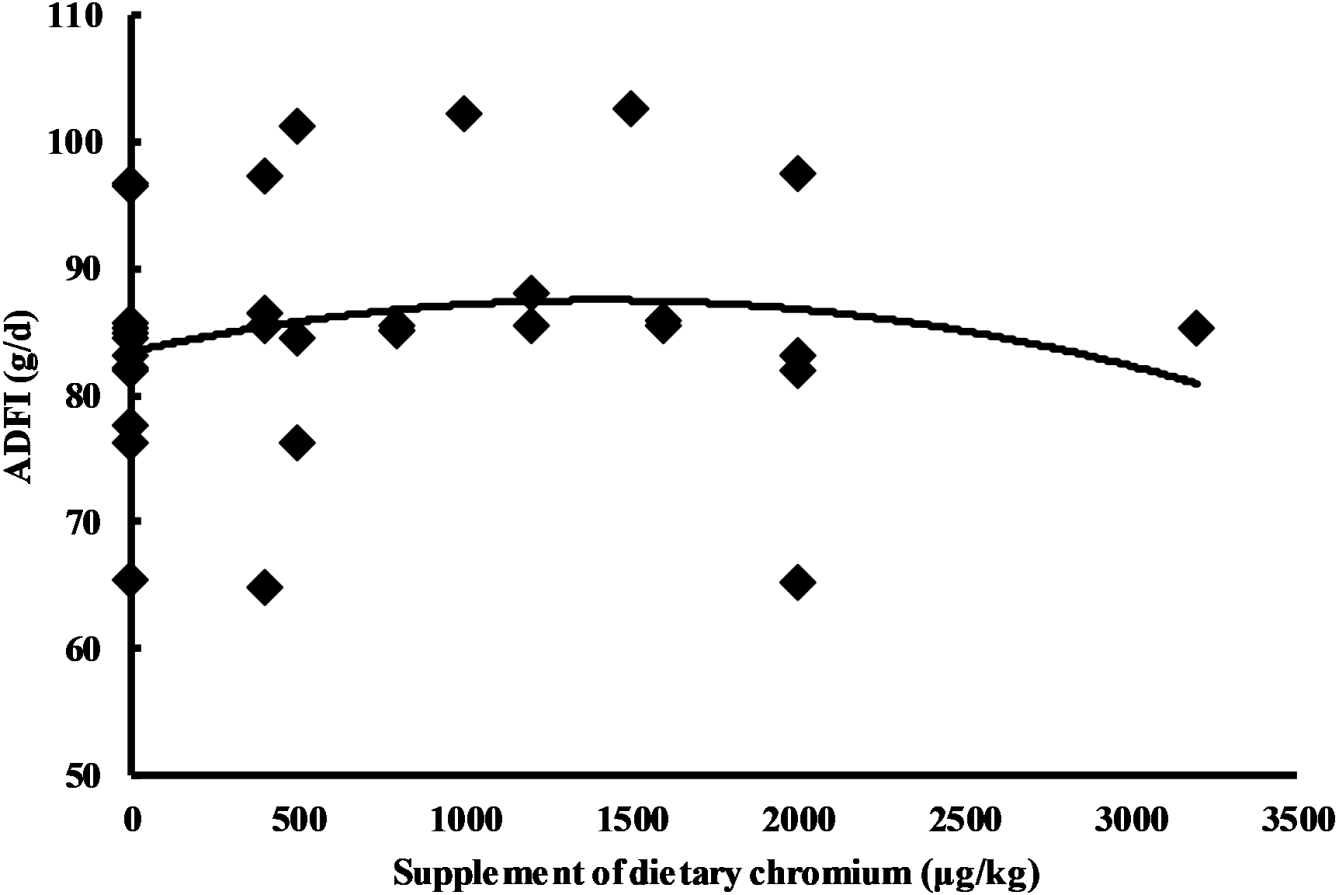
Relationship between chromium supplementation and adjusted ADFI.

**Figure 6:**
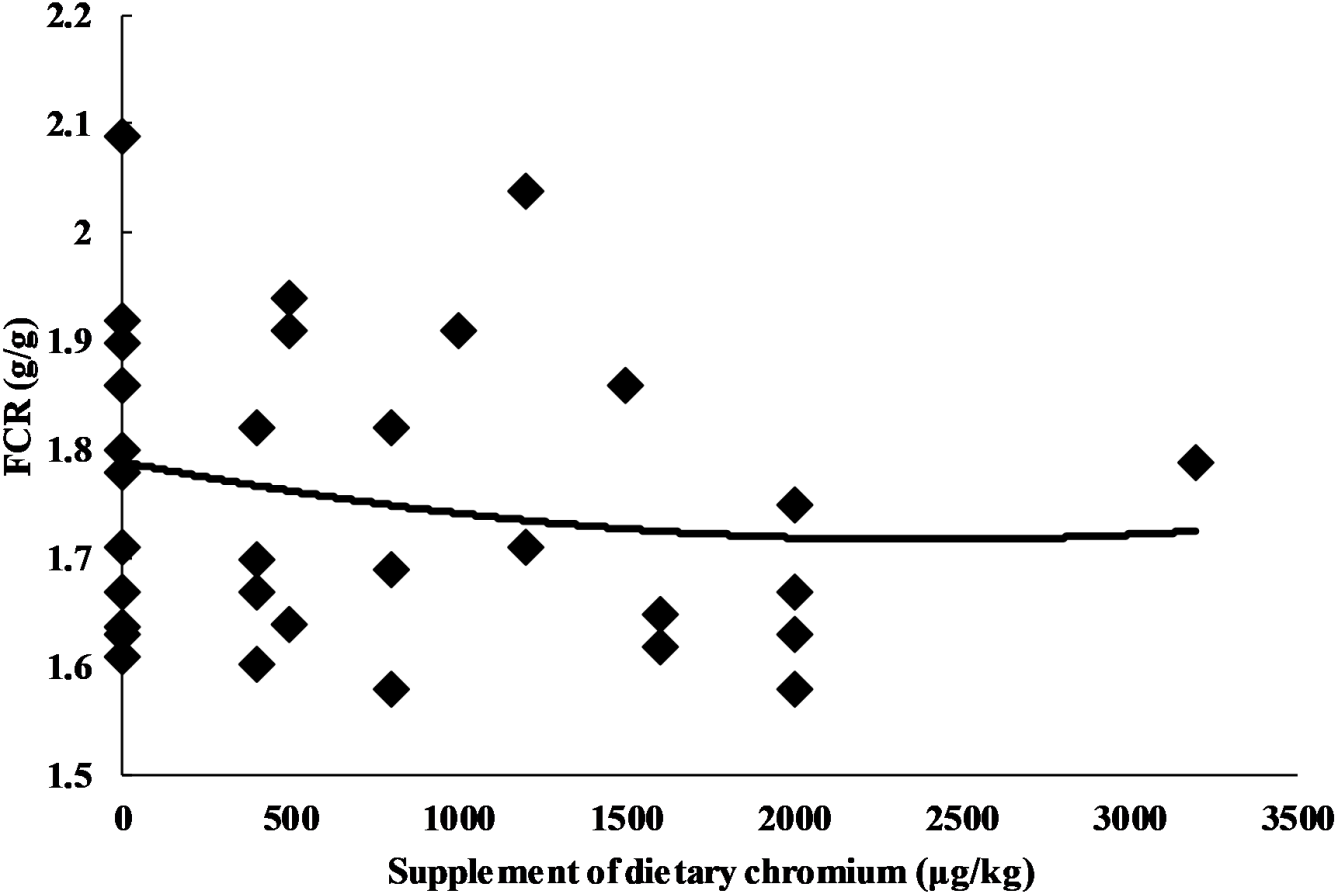
Relationship between chromium supplementation and adjusted FCR.

Chromium addition was used as the independent variable, and the performance indices (ADG, ADFI, and FCR) were used as the dependent variables. The result indicated a quadratic relationship between the adjusted ADFI and chromium addition (*Y*_ADFI_ = 84.022 + 1.839E−03X − 5.850E−07X^2^, *n* = 33, *P* = 0.026) (Table 2). The maximum value of the ADFI (88.358 g/d) was reached when chromium addition was 1.572 mg/kg (Fig. 5). Similarly, the result showed a quadratic relationship between the adjusted FCR and chromium addition (*Y*_FCR_ = 1.802 − 7.000E−05X + 1.508E−08X^2^, *n* = 33, *P* = 0.033) (Table 2). The minimum value of FCR (1.559 g/g) was reached when chromium addition as 2.321 mg/kg (Fig. 6). For ADG, our model may not be appropriate (maybe too complicated), and the results showed a lack of convergence (Table 2). In order to ensure the consistency of the model, we did not change the model.

**Table 1:** Summary of weighted mean differences from meta-analyses and stratified analyses by different factors.

	Stratification	No. of studies	Random effects model			Heterogeneity	
			WMD	95% CI	*P*	*I*^2^	*P*
**ADG**							
Main analysis		9	1.872	−0.376–4.121	0.103	99.5%	<0.001
Study area	China	4	0.410	−0.078–0.898	0.099	0%	0.469
	Iran	3	2.484	0.154–4.813	0.037	88.7%	<0.001
	Egypt	2	3.311	−1.321–7.942	0.161	99.8%	<0.001
Time	2000–2010	3	2.129	−2.199–6.457	0.335	99.5%	<0.001
	2010–2020	6	1.602	0.663–2.541	0.001	78.4%	<0.001
Sex	Both	3	3.578	−0.173–7.328	0.062	99.8%	<0.001
	Female	2	0.456	−0.590–1.501	0.393	0%	0.442
	Male	4	0.890	0.012–1.768	0.047	69.1%	0.021
Broiler strains	Hubbard	1	5.671	5.640–5.703	<0.001	–	–
	Cobb 500	3	0.910	0.629–1.191	<0.001	0%	0.503
	Arbor Acres	2	0.450	−0.339–1.239	0.263	48.3%	0.164
	Ross 308	3	2.484	0.154–4.813	0.037	88.7%	<0.001
**ADFI**							
Main analysis		9	1.023	0.217–1.828	0.013	58.7%	0.013
Study area	China	4	0.157	−0.646–0.960	0.701	0%	0.847
	Iran	3	2.461	−0.279–5.111	0.079	71.7%	0.029
	Egypt	2	1.363	1.329–1.397	<0.001	0%	0.333
Time	2000-2010	3	0.891	−0.164–1.946	0.098	42.1%	0.178
	2010-2020	6	1.308	−0.148–2.763	0.078	65.5%	0.013
Sex	Both	3	1.363	1.329–1.397	<0.001	0%	0.568
	Female	2	0.784	−0.927–2.494	0.369	0%	0.013
	Male	4	1.154	−0.955–3.262	0.284	77.8%	0.004
Broiler strains	Hubbard	1	1.363	1.329–1.397	<0.001	–	–
	Cobb 500	3	0.564	−0.909–2.036	0.453	0%	0.826
	Arbor Acres	2	−0.002	−0.929–0.889	0.966	0%	0.912
	Ross 308	3	2.416	−0.279–5.111	0.079	71.7%	0.029
**FCR**							
Main analysis		9	−0.060	−0.118—0.001	0.045	100%	<0.001
Study area	China	4	−0.040	−0.063—0.018	0.001	99.6%	<0.001
	Iran	3	−0.063	−0.175–0.049	0.267	100%	<0.001
	Egypt	2	−0.094	−0.219–0.031	0.141	100%	<0.001
Time	2000-2010	3	−0.063	−0.173–0.048	0.265	100%	<0.001
	2010-2020	6	−0.059	−0.120–0.003	0.063	100%	<0.001
Sex	Both	3	−0.116	−0.193 to −0.039	0.003	100%	<0.001
	Female	2	−0.055	−0.104 to −0.006	0.028	99.8%	<0.001
	Male	4	−0.021	−0.035 to −0.007	0.003	98.8%	<0.001
Broiler strains	Hubbard	1	−0.158	−0.158—0.157	<0.001	–	–
	Cobb 500	3	−0.047	−0.006—0.027	<0.001	99.7%	<0.001
	Arbor Acres	2	−0.025	−0.055–0.004	0.095	98.4%	<0.001
	Ross 308	3	−0.063	−0.175–0.049	0.267	100%	<0.001

**Table 2:** Regression relationships inferred using mixed effected models.

**Dependent variable**	**Independent variable**	**Intercept**	**SE**	***P*value**	**Quadratic**	**SE**	***P*value**	*R*^2^
ADFI	Cr addition	84.022	3.467	<0.001	−5.850E−07	<0.001	0.026	0.344
FCR			1.802	0.047	<0.001	1.508E−08	<0.001	0.033	0.368
ADG		46.478	0.994	<0.001	7.881E−07	<0.001	0.054	0.385

### Sensitivity analyses and publication bias

**Figure 7:**
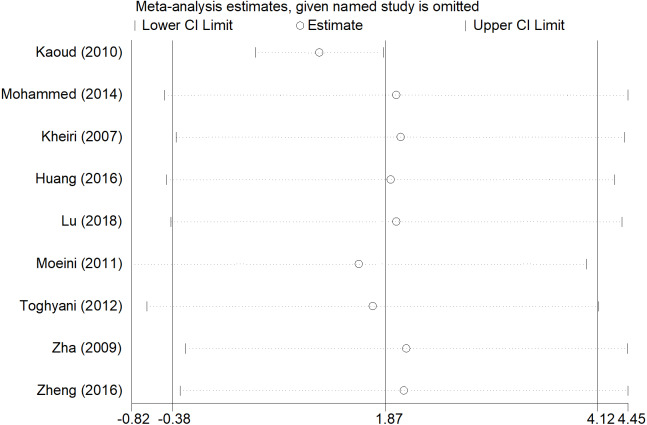
Sensitivity analysis of ADG.

**Figure 8:**
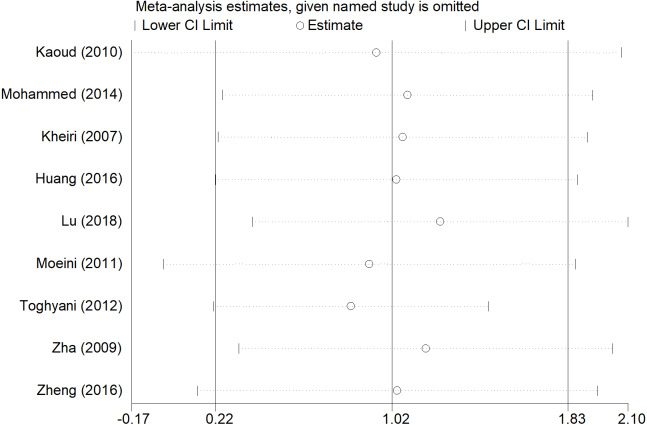
Sensitivity analysis of ADFI.

**Figure 9:**
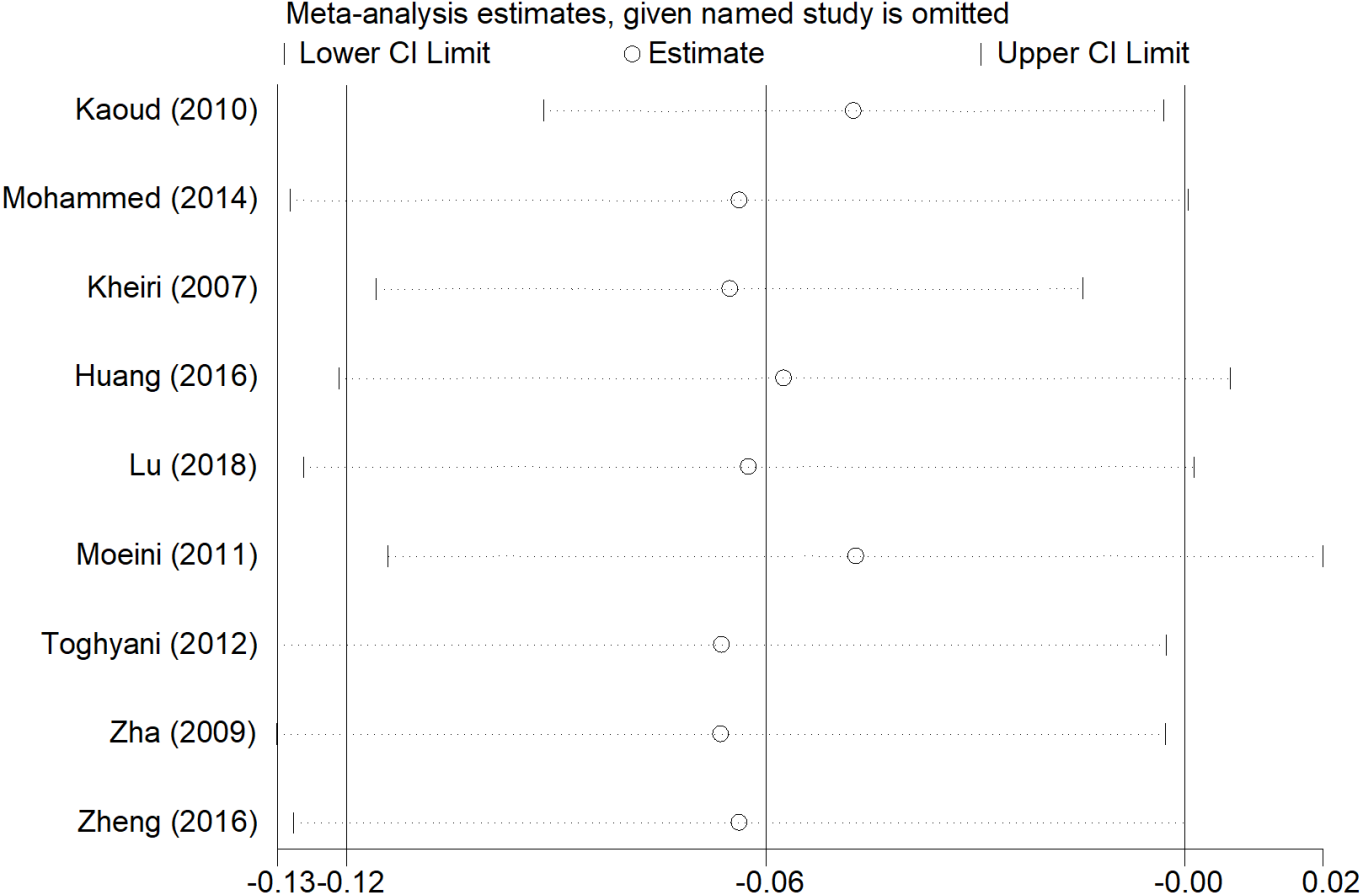
Sensitivity analysis of FCR.

After removing each study, the pooled estimates of the remaining studies were all located in the range of the overall effects, indicating that the meta-analysis results showed low sensitivity and high stability (Figs. 7, 8 and 9). There was little indication of publication bias for studies (*P* for Egger’s test was 0.212, 0.451, and 0.250, respectively).

## Discussion

Chromium was considered as an essential trace mineral for the boiler in manipulating growth performance and improving carcass composition. However, studies have different conclusions on the effect of chromium supplementation. Also, NRC did not specify the amount of chromium in poultry diets (NRC, 1997). Some researchers suggested that supplement of chromium at different levels in poultry improved feed intake and efficiency because of potentiation of insulin action, which shifts the overall metabolic responses more towards the anabolic side (Samanta, Haldar & Ghosh, 2008; Ahmed et al., 2005). Studies have also shown an adverse effect or no effect of chromium on feed intake, FCR, or body weight gain of broilers (Lu et al., 2019; Zha et al., 2009; Naghieh et al., 2010). This may be related to the lower bioavailability of inorganic chromium than organic chromium. On the other hand, Lu et al. suggested that the broiler’s chromium requirement might be higher under environmentally stressed conditions (Lu et al., 2019). Therefore, the growth performance of broilers was not significantly enhanced under normal environmental conditions.

The purpose of this study was to undertake a meta-analysis of trials assessing the effects of inorganic chromium supplementation on broiler growth performance and provide a more accurate dietary addition of inorganic chromium. To our knowledge, the present study is the first meta-analysis of the association between inorganic chromium dosage and ADG, ADFI, and FCR of broilers. A total of 9 studies that met our eligibility criteria were included in the meta-analysis  (Toghyani et al., 2012; Mohammed et al., 2014; Huang et al., 2016; Zheng et al., 2016; Lu et al., 2019; Zha et al., 2009; Kaoud, 2010; Kheiri & Toghyani, 2007; Moeini et al., 2011). By analyzing the data from 9 studies, we found that adding inorganic chromium had no significant effect on the ADG of broilers (Fig. 2) but significantly increased the ADFI and reduced the FCR of broilers (Figs. 3 and 4). The higher ADFI and lower FCR in this study agreed with early reports (Toghyani et al., 2012; Samanta, Haldar & Ghosh, 2008; Amatya, Haldar & Ghosh, 2004), and this may be attributed to the ability of chromium to regulate the level of glucose by activating insulin secretion and enable proper metabolic transformations of carbohydrates, proteins, and lipids (Haq et al., 2016; Chowdhury et al., 2003; Vincent, 2009). The result of ADG was in accord with previous studies reporting that dietary CrCl_3_ supplementation had little effect on broiler’s growth performance (Lu et al., 2019; Moeini et al., 2011; Ghazi et al., 2012). Ghazi et al. believed that dietary supplements of 0.6 mg/kg CrCl_3_ had no statistically significant effect on body mass and weight gain at 42 days of age (Ghazi et al., 2012). Even with the increase of chromium addition (0.8 or 1.2 mg/kg CrCl_3_), Moeini et al. (2011) reached the same conclusion as Ghazi et al.. Therefore, the effects of chromium supplementation on broiler weight gain, food intake, and feed conversion ratio are not consistent. We speculated that the reason might be related to heat stress. Stressors such as high environmental temperature increase the production and release of corticosteroids, affecting glucose and mineral metabolism, hypercholesterolemia, gastrointestinal lesions, alterations in immune system functions, and decreased tissue vitamin and mineral concentrations in poultry as well as humans (Siegel, 1995). Besides, the released corticosteroids increase chromium mobilization from tissues and its excretion by urinary output (Sahin & Sahin, 2002), which may exacerbate a marginal chromium deficiency or an increased chromium requirement. Previous studies conducted under heat stress conditions indicated that dietary chromium supplementation positively affected broiler growth performance  (Toghyani et al., 2012; Huang et al., 2016; Zha et al., 2009; Moeini et al., 2011), which may be directly related to chromium deficiency and increased chromium demand caused by corticosteroid release. Due to the lack of information from the included studies, no subgroup analysis of environmental stress conditions was conducted in this study. In other words, this meta-analysis could be influenced by stresses, such as temperature or cage area, which might considerably increase the heterogeneity of the data and lead to the fact that broiler’s weight gain had no significant change to the addition of inorganic chromium. Besides, the chromium source, administration level, background of the broiler strains, broiler age, and farm hygiene may be the reasons for the non-significance. However, under the premise that strict literature screening criteria had been established in this study, we tend to believe that environmental stress conditions might be the main factors.

Compared with the overall analyses of ADFI and FCR, our subgroup analyses produced more conservative effect estimates at country and published year group. Inorganic chromium addition was not associated with ADFI and FCR in the subgroup analyses, except for Egypt and China (Table 1). Although the overall analyses showed no significant association between ADG and inorganic chromium, subgroup analyses of country and published time indicated an inverse association in Iran and 2010s (Table 1). We believed that the broiler management practices and strategies were diverse in different areas and periods, which may lead to the inconsistent use of chromium and affect the broiler’s growth performance in the broiler industry. The subgroup analyses of ADG, ADFI, and FCR were consistent with the overall analyses regardless of broiler sex, indicating the high stability of meta-analysis results and then confirmed by sensitivity analyses (Figs. 7, 8 and 9). Besides, we found that male broiler chickens had better growth performance than females (higher ADG), which suggested that males might be more sensitive to chromium addition. Studies on different animals showed that chromium increased lean body mass (Page et al., 1993; Lindemann et al., 1995; Boleman et al., 1995), and the carcass lean rate of male broiler is always higher than that of female, which may be the reason for the higher ADG of male broiler chickens. The subgroup analyses stratified by broiler strains indicated no significant association between chromium and growth performance in Arbor Acres. Meanwhile, the result was difficult to estimate if only very few studies are included in Hubbard (Table 1). Compared with Ross 308, Cobb 500 had better growth performance (lower FCR) with inorganic chromium supplementation. Bjedov et al. reported that Cobb 500 showed lower FCR, higher performance index, and lower mortality than other broiler strain groups (Bjedov et al., 2011). Rosário et al. (2008) evaluated broiler performance under a canonical discriminant analysis and found a clear distinction between strains, where Cobb 500 presented the highest multivariate performance means. However, studies also found no statistically significant difference in body weight and feed conversion between Cobb 500 and Ross 308 (Kassab & Neemy, 2013; Shaheen et al., 2019), suggesting that researchers have not reached a consensus on different broiler strains’ growth performance. Thus, differences among broiler strains still cannot fully explain the reason why broilers showed different growth performance to inorganic chromium supplementation. We speculate that inorganic chromium has complicated effects on the physiological and metabolic process, whereas these are still poorly understood.

With the increase in chromium addition, the ADFI and FCR showed a significant negative and positive quadratic relationship, reaching an extreme value when chromium addition was 1.572 mg/kg and 2.321 mg/kg, respectively (Figs. 5 and 6). A lack of convergence indicated that the model did not fit the data of ADG, the reason of which might be that the model was too complicated. However, we did not modify the model in order to ensure consistency. The amount of chromium addition has always been controversial in the poultry industry. Kaoud reported that 0.2 mg/kg CrCl_3_ supplementation improved aspects of growth performance, carcass traits, and immune response (Kaoud, 2010), while Lu et al. believed that 3.2 mg/kg dietary Cr supplementation did not affect on average daily gain, average daily feed intake and, gain: feed of broilers during the starter and grower periods (Lu et al., 2019). This meta-analysis found that the growth performance might be improved with the inorganic chromium addition ranging from 1.6 mg/kg to 2.3 mg/kg. This result is similar to an experimental study reporting that 2.0 mg/kg dietary inorganic chromium supplementation significantly improved broiler’s growth performance (Norain et al., 2013). We tend to consider 2.3 mg/kg as the appropriate additional amount because the FCR reached a minimum at this point. The feed constitutes 70%–80% of the cost in raising broiler chickens. Therefore, changes in FCR can have a significant impact on the profitability of an operation (Aggrey et al., 2010). According to the positive quadratic relationship between FCR and chromium addition, the FCR gradually increased after higher than 2.3 mg/kg. It is worth noting that the broiler’s FCR may not change significantly even with high inorganic chromium supplementation (3.2 mg/kg) as mentioned above (Lu et al., 2019), which may indicate that there was not a simple quadratic relationship between inorganic chromium and broiler performance. Hence, more experimental data are needed to support and explain this speculation.

The literature sources greatly influence meta-analysis, however, all the studies we selected were from English databases. Paper published in other languages, conference proceedings, and some unpublished results may have an impact on this meta-analysis. In addition, the number of studies included was limited. Although there was no publication bias in these studies, only one study was included in some subgroups, which does not lead to robust results. Finally, the dose range of chromium was narrow, and the model may change when pharmacologically relevant doses rather than nutritionally relevant doses are used. Thus, the results of this paper may have some limitations.

## Conclusions

A meta-analysis is a statistical analysis that combines the results of multiple scientific studies. The effect of inorganic chromium supplementation on broiler growth performance has always been controversial. Through meta-analysis, our study showed that the males and Cobb 500 broilers might be more sensitive to chromium supplementation and provided more accurate inorganic chromium supplementation for the management practice of broiler. However, this study still has some limitations. The fewer included studies may lead to higher heterogeneity and thus affect the results of the meta-analysis. Additionally, no subgroup analysis of environmental stress conditions was conducted due to the lack of related information. We expect more experimental studies and systematic reviews to provide support and explanation for the response mechanism of broiler performance to feed additives.

##  Supplemental Information

10.7717/peerj.11097/supp-1 Table S1 The characteristics of Meta-analysis database

10.7717/peerj.11097/supp-2 Table S2 PRISMA checklist

10.7717/peerj.11097/supp-3 Table S3 Raw data

10.7717/peerj.11097/supp-4 Supplemental Information 1 Meta-Analysis Rationale

10.7717/peerj.11097/supp-5 Supplemental Information 2 Detailed search strategy

## Additional Information and Declarations

### Data Availability

The following information was supplied regarding data availability: Raw data are available in the Supplemental Files.
