# Supplementary material for: Effects of dietary inorganic chromium supplementation on broiler growth performance: a meta-analysis
Source: PeerJ. 2021 Mar 16;9:e11097. doi: 10.7717/peerj.11097 (PMC7977379; doi:10.7717/peerj.11097)
Supplement: Table S1 [file peerj-09-11097-s001.docx]

**Table S1 The characteristics of Meta-analysis database**

| **Author** | **Country** | **Published Year** | **Strains** | **Sex** | **Basal diet** | **Chromium source** | **Cr addition (μg/kg)** | **Sample Size** | **Experimental period (d)** |
| --- | --- | --- | --- | --- | --- | --- | --- | --- | --- |
| Toghyani [7] | Iran | 2012 | Ross 308 | Male | corn+soybean | CrCl_3_·6H_2_O | 0, 500, 1000, 1500 | 420 | 42 |
| Mohammed [8] | Egypt | 2014 | Cobb 500 | Both | corn+soybean | CrCl_3_·6H_2_O | 0, 500 | 225 | 42 |
| Huang [9] | China | 2016 | Cobb 500 | Female | corn+soybean | CrCl_3_·6H_2_O | 0, 400, 2000 | 252 | 42 |
| Zheng [11] | China | 2016 | Cobb 500 | Female | corn+soybean | CrCl_3_·6H_2_O | 0, 400, 2000 | 252 | 42 |
| Lu [13] | China | 2018 | Arbor Acres | Male | corn+soybean | CrCl_3_·6H_2_O | 0, 400, 800, 1600, 3200 | 432 | 42 |
| Zha [14] | China | 2009 | Arbor Acres | Male | corn+soybean | CrCl_3_·6H_2_O | 0, 500 | 240 | 42 |
| Kaoud [22] | Egypt | 2010 | Hubbard | Both | corn+soybean | CrCl_3_·6H_2_O | 0, 2000 | 2700 | 42 |
| Kheiri [23] | Iran | 2007 | Ross 308 | Male | corn+soybean | CrCl_3_·6H_2_O | 0, 400, 800 1200, 1600 | 300 | 42 |
| Moeini [24] | Iran | 2011 | Ross 308 | Both | corn+soybean | CrCl_3_·6H_2_O | 0, 800, 1200 | 250 | 42 |
