# Supplementary material for: Effects of dietary inorganic chromium supplementation on broiler growth performance: a meta-analysis
Source: PeerJ. 2021 Mar 16;9:e11097. doi: 10.7717/peerj.11097 (PMC7977379; doi:10.7717/peerj.11097)
Supplement: Supplemental Information 1 [file peerj-09-11097-s004.docx]

**Meta-Analysis Rationale**

**1. The rationale for conducting this meta-analysis:**

Chromium (Cr), as an essential trace element, plays an important role in insulin action. and promote the efficiency of glucose, protein and fat metabolism, which was also recognized as a dietary supplement by human beings. Among inorganic sources, the hexavalent chromium is a known toxin, mutagen, and carcinogen, whereas, trivalent chromium is the stable oxidation state and is considered to be a highly safe form which play an important role in improving broiler growth performance.

However, there are no NRC recommendations for chromium in broiler diets currently and the efficacy of wildly used inorganic chromium in broiler industry has not been fully researched. Previous studies reported that chromium could increase weight gain, feed conversion ratio, high density lipoprotein and improve lean muscle development and nutrient digestion. The beneficial effects of chromium can be observed more efficiently under environmental, dietary and hormonal stress. Contradictorily, some studies showed that inorganic chromium supplementation in broiler diet had no effect on growth performance. Thus, the effect of chromium addition on broiler growth performance has not been concluded.

The main goal of this study is to explore the relationship between inorganic chromium addition and broiler performance reported by different studies through meta-analysis and obtain a reasonable quantitative model to explain the observed value and provide a theoretical reference for the practical process.

**2. The contribution that the meta-analysis makes to knowledge in light of previously published related reports, including other meta-analyses and systematic reviews:**

This is the first meta-analysis for effects of dietary inorganic chromium supplementation on broiler growth performance. Our study demonstrated that inorganic chromium had a positive effect on the growth performance of broilers. Compared with the previous amount of inorganic chromium, we limited the dose in a more accurate range, which is of great significance to the practice of broiler industry.
