# Supplementary material for: Effects of dietary inorganic chromium supplementation on broiler growth performance: a meta-analysis
Source: PeerJ. 2021 Mar 16;9:e11097. doi: 10.7717/peerj.11097 (PMC7977379; doi:10.7717/peerj.11097)
Supplement: Supplemental Information 2 [file peerj-09-11097-s005.docx]

**Detailed search strategy:**

(broiler OR chick*) AND (performance OR growth) AND (chromium chloride or inorganic chromium)
